# RNA profiling of cyclooxygenases 1 and 2 in colorectal cancer

**DOI:** 10.1038/sj.bjc.6602119

**Published:** 2004-08-24

**Authors:** R D Church, J Yu, J W Fleshman, W D Shannon, R Govindan, H L McLeod

**Affiliations:** 1 Department of Surgery, Washington University School of Medicine, St Louis, MO 63110-1093, USA; 2 Department of Medicine, Washington University School of Medicine, 660 South Euclid Ave, Campus Box 8069, St Louis, MO 63110-1093, USA; 3 The Siteman Cancer Center, Washington University School of Medicine, St Louis, MO 63110-1093, USA; 4 Division of Biostatistics, Washington University School of Medicine, St Louis, MO 63110-1093, USA; 5 Department of Genetics, Washington University School of Medicine, St Louis, MO 63110-1093, USA; 6 Department of Molecular Biology and Pharmacology, Washington University School of Medicine, St Louis, MO 63110-1093, USA

**Keywords:** cyclooxygenase, colorectal cancer, gene expression

## Abstract

Cyclooxygenases (particularily Cox-2) are involved in carcinogenesis and metastatic cancer progression. The expression profiles of the cyclooxygenases and the roles they play in established tumours of similar stage remains unclear. We report that Cox-1 and Cox-2 expression is highly variable in Dukes' C tumours, and changes in Cox-1 expression may be of importance.

Colorectal cancer is the third most common cancer in the Western world and despite advances in surgery, adjuvant therapies and screening, little impact on the mortality rates has been seen (Jemal *et al*, 2003). A greater understanding of the molecular mechanisms underlying carcinogenesis and progression is leading to novel treatment strategies.

Cyclooxygenases (Cox) are responsible for the metabolism of arachidonic acid into prostaglandins (Church *et al*, 2003). Two isoforms exist, termed Cox-1 and Cox-2 (Vane, 1971; Xie *et al*, 1991). Increased expression of Cox-2 has been implicated in carcinogenesis and metastatic progression in many forms of human cancer (Church *et al*, 2003). For example, increased expression of Cox-2 protein has been shown to correlate with tumour invasiveness and metastasis (Chen *et al*, 2001). In addition, 100% of metastatic lesions had positive immunohistochemical staining for Cox-2 *vs* 72% of primary tumours (Zhang and Sun, 2002). Cox-2 inhibitors are now being evaluated as adjuncts to chemotherapy for colon cancer (Blanke, 2002; Blanke and Masferrer, 2003).

Initial evidence with regard to the expression of Cox-1 suggested a minimal role in colonic neoplasia, with several studies demonstrating minimal expression of Cox-1 with little variability in polyps and established tumours (Eberhart *et al*, 1994; Sano *et al*, 1995). More recent evidence suggests that Cox-1 expression and activity may have a role to play in the carcinogenic process (Takeda *et al*, 2003). For example, reduced polyp formation was seen in MIN mice lacking a functional Cox-1 gene (Chulada *et al*, 2000) and Cox-1 expression may promote carcinogenesis in lung and gynaecological tissues, both synergistically with and independently of Cox-2 (Hasturk *et al*, 2002; Sales *et al*, 2002; Gupta *et al*, 2003). We used real time PCR to investigate Cyclooxygenase 1 and 2 expression profiles in invasive colonic tumours. Our aims were to define cyclooxygenase expression patterns in established colorectal tumours compared to adjacent normal mucosa and correlate this with clinicopathological variables and patient outcome.

## MATERIALS AND METHODS

### Patients

In total, 51 stage III (Dukes' C) colorectal cancer patients had tumour and adjacent normal bowel mucosa samples collected at the time of surgical resection by The Siteman Cancer Center Tissue Procurement Core. The median age of these patients was 68 (range 39–96 years). All samples were snap frozen and stored at −80°C until used for RNA extraction. In total, 29 patients (56.9%) were male. Approval for this study, including the genomic analysis of the tissue samples, was obtained from the Washington University in St Louis Human Studies Committee. All patients gave informed consent. Clinical data were collected prospectively and used to compare expression with tumour differentiation, anatomic location (either left or right colon), survival, recurrence (both metastatic and local recurrence), patient gender and age.

### RNA extraction and real time PCR for cyclooxygenases

The TRIzol RNA isolation kit (Invitrogen, Carlsbad, CA, USA) was used for RNA extraction from the paired tumour and normal mucosa. Areas of high cellularity on light microscopy (median 86%, range 65–95%) were chosen from each tissue sample. RNA was quantified and assessed for purity by measurement of OD260 and OD280 using a UV fiberoptic spectrophotometer (Nanodrop Technologies, Rockland, DE, USA) and was qualitatively assessed by measurement of relative 28S and 18S ribosomal band intensities using a Bioanalyzer and RNA NanoChip capillary gel electrophoresis assay (Agilent Technologies, Palo Alto, CA, USA). RNAs were reverse-transcribed into cDNA samples using Superscript II reverse transcriptase (Invitrogen, Carlsbad, CA, USA). Primers and probes for the Real Time PCR for Cox-1 and Cox-2 RNA were designed using the Primer Express Software (ABI, Foster City, CA, USA) (Table 1

**Table 1:** RT–PCR probes for Cox-1, Cox-2 and the reference gene APP. The fluorophores FAM, JOE, TAMRA were used, as indicated

**Gene**	**Primer**	**Primer or probe sequence**
Cox-1	Probe	5′-FAM/CCTTCCATCCGGTGGCCTATTCCA/36-TAMRA
	Forward primer	5′-CTGCCCTCCTCAAGACTTTAGCTT
	Reverse primer	5′-TCCAACTGATTTAAGCAAAAGAGGAAT
Cox-2	Probe	5′-FAM/AATCAAGCCTGGCTAGCATGCTG/36-TAMRA
	Forward primer	5′-TGAAGCCAATTCAGTAGGTGCAT
	Reverse primer	5′-ATCGCTAAAAGAAAAGAAAAGGA
		
APP reference gene	Probe	5′-JOE/AGTTCAGCCTGGACGATCTCCAGCC/TAMRA
	Forward primer	5′-CTCATGCCATCTTTGACCGA
	Reverse primer	5′-GGGCATCAACAGGCTCAACT

). The probe and primer sets were synthesised by Integrated DNA Technologies (Coralvile, IA, USA). The relative RNA quantitation was assessed by Taqman real time PCR using an ABI PRISM 7700 analyser (Applied Biosystems, Foster City, CA, USA). All real time PCR assays were performed in triplicate.

### Measurement of relative RNA expression levels

The relative expression levels were calculated using the modified comparative CT method (Pfaffl, 2001). The PCR efficiencies were calculated from standard curves using the formula *E*=10[−1/slope] where *E* is the efficiency and slope is the slope of the standard curve. Standard curves for the reference and cyclooxygenase genes constituted separate experiments using pooled colorectal cancer RNA samples (data not shown). The APP gene was used as the internal reference. The relative expression level of the RNA for each Cox gene was normalised to the APP gene and to one of all of the 102 tissue samples. The calibrator sample chosen was that which had the maximum *C*_T_ value, that is, the lowest expression level. The normalised relative expression levels for each gene was calculated using the following formula (Pfaffl, 2001):


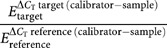


where *E*_target_ is the real-time PCR frequency of the target gene transcript and *E*_reference_ is the real time PCR efficiency of the reference gene transcript.

### Statistics

Statistical analyses were performed using GraphPad InStat version 3.05 for GraphPad Software (San Diego, CA USA). Wilcoxon matched pairs test and Spearman's Rank Correlation coefficient were used to evaluate the differences seen in expression levels of the Cox enzymes between the samples. Kruskal–Wallis Test (Nonparametric ANOVA) and Mann–Whitney *U* test was used to compare cyclooxygenase expression and clinical and pathological variables. Kaplan–Meier analyses were carried out when comparing survival times. The *P*-values of <0.05 were considered to be significant.

## RESULTS

**Figure 1:**
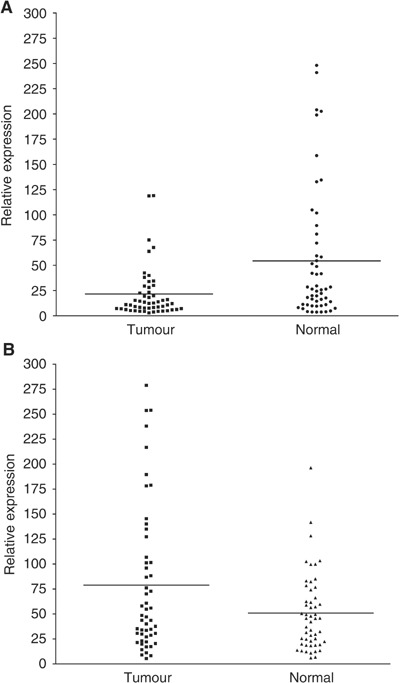
Comparison of variations in expression of Cox 1 (**A**) and Cox 2 (**B**) paired normal mucosa and tumour tissue samples. Horizontal lines represent the mean for each population.

Substantial variation in the expression of Cyclooxygenase 2 mRNA was observed in normal mucosa (33-fold) and tumour tissues (51-fold). Variable Cox-1 expression was also seen in normal mucosa (68-fold) and tumour (40-fold). Cox-2 was significantly upregulated in the tumour samples compared to paired mucosal tissues (median tumour : normal ratio=1.54, range 0.20–8.96, *P*=0.012, Figure 1B

). In contrast, tumour Cox-1 expression was significantly lower than normal mucosal samples (median tumour : normal ratio=0.48, range 0.01–2.85, *P*<0.0001, Figure 1A). The expression levels of each enzyme in normal mucosa also correlated to the expression seen in paired malignant mucosa (Cox-1, *r*_s_=0.63, *P*<0.0001; Cox-2, *r*_s_=0.33, *P*=0.008).

Cyclooxygenase-2 expression in tumour tissues did not correlate with disease recurrence (*P*=0.16), tumour differentiation (*P*=0.26), gender (*P*=0.2), age >70 (*P*=0.06), or site of tumour (*P*=0.84). Cox-1 expression similarly did not show any significantly different expression in tumour or normal mucosa in relation to these clinicopathological variables.

**Figure 2 fig2:**
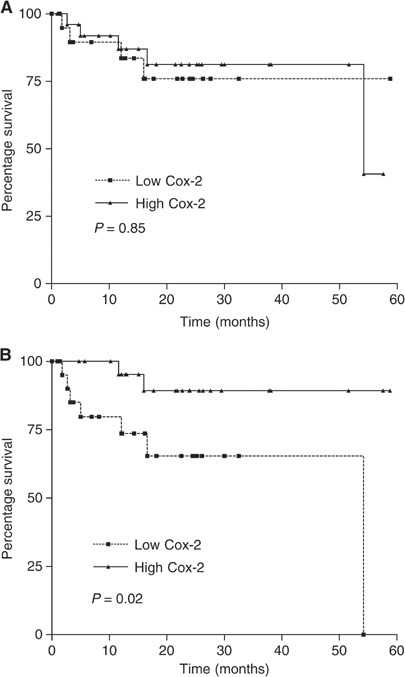
Relationship between patient survival and Cox-2 expression in tumour tissues (**A**) and normal mucosa (**B**).

The relationships between Cox-1 and Cox-2 were also examined ([Fig fig2]

Figure 3A and B

**Figure 3:**
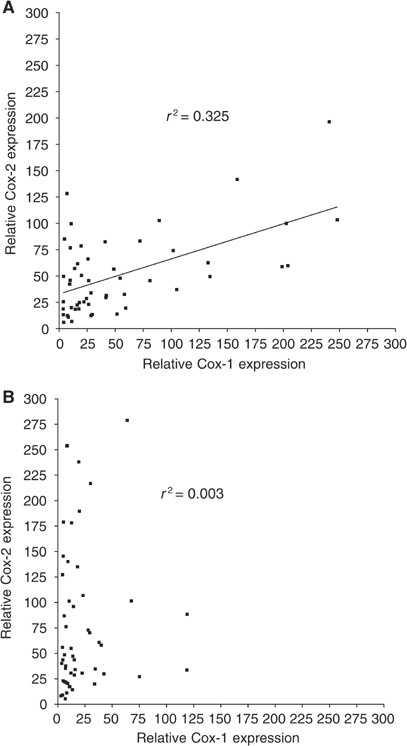
Relationship between Cox-1 and Cox-2 expression in normal mucosa (**A**) and tumour (**B**).

). In normal tissue a linear relationship could be seen between Cox-1 and Cox-2 expression (Figure 3A, *r*^2^=0.32). However this relationship was clearly not maintained in the tumour tissues (Figure 3B, *r*^2^=0.003) with an increased expression of Cox-2 protein relative to Cox-1 expression.

Patients were divided into high or low expression groups using the median expression values for each cyclooxygenase gene. No significant differences in cancer-specific survival were seen using Cox-1 expression in normal (*P*=0.26) or malignant tissues (*P*=0.36). Cox-2 expression in the tumour did not correlate with survival (*P*=0.85, Figure 2A) but patients expressing high levels of Cox-2 in the normal mucosa appeared to have a survival advantage (*P*=0.02; Figure 2B).

## DISCUSSION

Cox-1 and Cox-2 expression seen in normal and malignant mucosa showed wide variation, even in the context of patients with the same clinical disease stage. The validity of such variation could be confirmed with immunohistochemistry but the recovery of tissue slides for inclusion in this pilot study was not possible. The previously reported immunohistochemical studies have also shown large differences in the staining intensity, and the numbers of cells expressing the Cox-2 protein (Hao *et al*, 1999; Masunaga *et al*, 2000; Cianchi *et al*, 2001). Our RNA expression data highlight such previously observed variability. These and other studies have been able to show that such elevated expression of Cox-2 correlated with clinicopathological variables. However, thresholds for positivity in these studies were low, including cells weakly stained, and sections with less than 10% of epithelial cell population deemed to be positive (Cianchi *et al*, 2001; Zhang and Sun, 2002). In addition, these studies utilised samples obtained across various disease stages. The increased expression of cyclooxygenase-2 mRNA in tumour in this study is consistent with these previous studies (Church *et al*, 2003).

A direct molecular basis for the upregulation of Cox-2 in polyps and cancer is still poorly defined. However, one mechanism may be the clonal expansion of tumour cells that express Cox-2. Such increased expression seems to increase tumour angiogenesis and decrease cellular apoptosis, leading to improved overall cellular viability compared to tumours not aberrantly expressing this protein (Church *et al*, 2003).

We were not able to show differences in cancer-specific survival or disease recurrence in patients expressing high levels of Cox-2 in tumour. This may reflect the fact that our samples are from a well-defined stage of disease progression, that is, Dukes' C tumours. Previously, it has been suggested that Cox-2 expression is associated with poorer outcomes; however, these studies compared expression across clinical disease stages and were not able to demonstrate any predictive potential independent of Dukes' stage (Sheehan *et al*, 1999; Masunaga *et al*, 2000). Patients with a high level of Cox-2 expression in the normal mucosa did seem to have survival advantage. The reasons for this observation are not easily explained and conflicts with some previous studies that examined the expression of the cyclooxygenases in the malignant tumour (Church *et al*, 2003).

The expression levels of Cox-1 also demonstrated considerable variation in RNA expression in normal and malignant tissues. This contrasts with the previously accepted opinion that Cox-1 exists as a house keeping gene, which is not subject to variable expression (Sano *et al*, 1995). More recent evidence suggests that Cox-1 is inducible and can be upregulated in malignant tissues (Sales *et al*, 2002; Gupta *et al*, 2003). We have shown that Cox-1 seemed to be downregulated in colorectal tumour specimens. Indeed, a synergistic relationship of the cyclooxygenases in the early stages of carcinogenesis has been suggested, with Cox-1 having a role initially followed by a rise in Cox-2 expression as the malignant process continues (Takeda *et al*, 2003). Our data confirm an altered regulation of Cox-1 expression between normal and malignant tissues, consistent with such suggestions. It has also been suggested that the increases of Cox-2 expression and the tissue-specific prostaglandin E Synthetase often seen in malignant tissue may be dependent on the expression of Cox-1, at least initially (Takeda *et al*, 2003). There is emerging evidence that Cox-1 may have a role to play in carcinogenesis in other solid tumours such as ovarian (Gupta *et al*, 2003) and skin cancer (Tiano *et al*, 2002). This may mean that the nonspecific cyclooxygenase inhibitors, such as sulindac and aspirin, may be more important agents in the prevention of colonic polyps, if compared to the Cox-2 specific inhibitors, such as celecoxib and rofecoxib, which are currently being studied in this context. However, the reduction in Cox-1 expression in more advanced disease supports the view that as additions to adjuvant therapy regimes specific Cox-2 inhibitors should be more effective.

## References

[bib3] Blanke CD (2002) Celecoxib with chemotherapy in colorectal cancer. Oncology (Huntingt) 16: 17–21 12014863

[bib4] Blanke CD, Masferrer JL (2003) Chemotherapy with cyclooxygenase-2 inhibitors in the treatment of malignant disease: pre-clinical rationale and preliminary results of clinical trials. Prog Exp Tumor Res 37: 243–260 12795058 10.1159/000071376

[bib5] Chen WS, Wei SJ, Liu JM, Hsiao M, Kou-Lin J, Yang WK (2001) Tumor invasiveness and liver metastasis of colon cancer cells correlated with cyclooxygenase-2 (COX-2) expression and inhibited by a COX-2-selective inhibitor, etodolac. Int J Cancer 91: 894–899 11275997 10.1002/1097-0215(200102)9999:9999<894::aid-ijc1146>3.0.co;2-#

[bib6] Chulada PC, Thompson MB, Mahler JF, Doyle CM, Gaul BW, Lee C, Tiano HF, Morham SG, Smithies O, Langenbach R (2000) Genetic disruption of Ptgs-1, as well as Ptgs-2, reduces intestinal tumorigenesis in Min mice. Cancer Res 60: 4705–4708 10987272

[bib7] Church RD, Fleshman JW, McLeod HL (2003) Cyclo-oxygenase 2 inhibition in colorectal cancer therapy. Br J Surg 90: 1055–1067 12945071 10.1002/bjs.4297

[bib8] Cianchi F, Cortesini C, Bechi P, Fantappie O, Messerini L, Vannacci A, Sardi I, Baroni G, Boddi V, Mazzanti R, Masini E (2001) Up-regulation of cyclooxygenase 2 gene expression correlates with tumor angiogenesis in human colorectal cancer. Gastroenterology 121: 1339–1347 11729113 10.1053/gast.2001.29691

[bib9] Eberhart CE, Coffey RJ, Radhika A, Giardiello FM, Ferrenbach S, DuBois RN (1994) Up-regulation of cyclooxygenase 2 gene expression in human colorectal adenomas and adenocarcinomas. Gastroenterology 107: 1183–1188 7926468 10.1016/0016-5085(94)90246-1

[bib10] Gupta RA, Tejada LV, Tong BJ, Das SK, Morrow JD, Dey SK, DuBois RN (2003) Cyclooxygenase-1 is overexpressed and promotes angiogenic growth factor production in ovarian cancer. Cancer Res 63: 906–911 12615701

[bib11] Hao X, Bishop AE, Wallace M, Wang H, Willcocks TC, Maclouf J, Polak JM, Knight S, Talbot IC (1999) Early expression of cyclo-oxygenase-2 during sporadic colorectal carcinogenesis. J Pathol 187: 295–301 10398082 10.1002/(SICI)1096-9896(199902)187:3<295::AID-PATH254>3.0.CO;2-Y

[bib12] Hasturk S, Kemp B, Kalapurakal SK, Kurie JM, Hong WK, Lee JS (2002) Expression of cyclooxygenase-1 and cyclooxygenase-2 in bronchial epithelium and nonsmall cell lung carcinoma. Cancer 94: 1023–1031 11920472

[bib13] Jemal A, Murray T, Samuels A, Ghafoor A, Ward E, Thun MJ (2003) Cancer statistics, 2003. CA Cancer J Clin 53: 5–26 12568441 10.3322/canjclin.53.1.5

[bib15] Masunaga R, Kohno H, Dhar DK, Ohno S, Shibakita M, Kinugasa S, Yoshimura H, Tachibana M, Kubota H, Nagasue N (2000) Cyclooxygenase-2 expression correlates with tumor neovascularization and prognosis in human colorectal carcinoma patients. Clin Cancer Res 6: 4064–4068 11051257

[bib16] Pfaffl MW (2001) A new mathematical model for relative quantification in real-time RT–PCR. Nucleic Acids Res 29: e45 11328886 10.1093/nar/29.9.e45 PMC55695

[bib17] Sales KJ, Katz AA, Howard B, Soeters RP, Millar RP, Jabbour HN (2002) Cyclooxygenase-1 is up-regulated in cervical carcinomas: autocrine/paracrine regulation of cyclooxygenase-2, prostaglandin e receptors, and angiogenic factors by cyclooxygenase-1. Cancer Res 62: 424–432 11809691 PMC2694304

[bib18] Sano H, Kawahito Y, Wilder RL, Hashiramoto A, Mukai S, Asai K, Kimura S, Kato H, Kondo M, Hla T (1995) Expression of cyclooxygenase-1 and -2 in human colorectal cancer. Cancer Res 55: 3785–3789 7641194

[bib19] Sheehan KM, Sheahan K, O'Donoghue DP, MacSweeney F, Conroy RM, Fitzgerald DJ, Murray FE (1999) The relationship between cyclooxygenase-2 expression and colorectal cancer. JAMA 282: 1254–1257 10517428 10.1001/jama.282.13.1254

[bib22] Takeda H, Sonoshita M, Oshima H, Sugihara K, Chulada PC, Langenbach R, Oshima M, Taketo MM (2003) Cooperation of cyclooxygenase 1 and cyclooxygenase 2 in intestinal polyposis. Cancer Res 63: 4872–4877 12941808

[bib23] Tiano HF, Loftin CD, Akunda J, Lee CA, Spalding J, Sessoms A, Dunson DB, Rogan EG, Morham SG, Smart RC, Langenbach R (2002) Deficiency of either cyclooxygenase (COX)-1 or COX-2 alters epidermal differentiation and reduces mouse skin tumorigenesis. Cancer Res 62: 3395–3401 12067981

[bib24] Vane JR (1971) Inhibition of prostaglandin synthesis as a mechanism of action for aspirin-like drugs. Nat New Biol 231: 232–235 5284360 10.1038/newbio231232a0

[bib25] Xie WL, Chipman JG, Robertson DL, Erikson RL, Simmons DL (1991) Expression of a mitogen-responsive gene encoding prostaglandin synthase is regulated by mRNA splicing. Proc Natl Acad Sci USA 88: 2692–2696 1849272 10.1073/pnas.88.7.2692 PMC51304

[bib26] Zhang H, Sun XF (2002) Overexpression of cyclooxygenase-2 correlates with advanced stages of colorectal cancer. Am J Gastroenterol 97: 1037–1041 12003384 10.1111/j.1572-0241.2002.05625.x

